# Ecofriendly single-step HPLC and TLC methods for concurrent analysis of ternary antifungal mixture in their pharmaceutical products

**DOI:** 10.1186/s13065-023-01083-1

**Published:** 2023-12-01

**Authors:** Maha M. Abdelrahman, Ibrahim A. Naguib, Hala E. Zaazaa, Hend M. Nagieb

**Affiliations:** 1 Pharmaceutical Analytical Chemistry Department, Faculty of Pharmacy, Beni-Suef University, Alshaheed Shehata Ahmad Hegazy St., Beni-Suef, 62514 Egypt; 2 Department of Pharmaceutical Chemistry, College of Pharmacy, Taif University, P.O. Box 11099, 21944 Taif, Saudi Arabia; 3 Pharmaceutical Analytical Chemistry Department, Faculty of Pharmacy, Cairo University, Kasr El-Aini St., Cairo, 11562 Egypt; 4 Pharmaceutical Chemistry Department, Faculty of Pharmacy, Nahda University, Beni-Suef, Egypt

**Keywords:** Miconazole nitrate, Nystatin, Metronidazole, HPLC–DAD, TLC, Green assessment, Method validation

## Abstract

Two accurate, sensitive, and selective methods for simultaneous determination of miconazole nitrate (MIC), nystatin (NYS), and metronidazole (MET) in pure state or drug product were established and verified. First, RP-HPLC-DAD was designed. Separation was accomplished using a ZOBRAX Eclipse Plus RP-C_8_ column that was running under an isocratic elution of methanol: 0.05% aqueous solution of sodium dodecyl sulphate (40: 60 v/v), with a flow rate that was regulated at 0.8 mL/min. The column temperature was adjusted at 25 °C and diode array detector was monitored at 220 nm. The linearity range of the proposed method was achieved at the concentration of 5–50, 4–50, and 4–40 µg/mL and the attained retention time for the studied drugs was 2.52, 3.52 and 4.99 min for MIC, NYS, and MET, correspondingly. Second, a TLC-densitometric approach was used to resolve the three compounds. Resolution of the three cited drugs was carried out using TLC aluminum plates pre-coated with 0.25 mm silica gel 60 F_254_. A developing solvent comprised ethyl acetate: toluene: methanol: triethyl amine: formic acid (3: 1: 7: 0.3: 0.1 by volume) (pH = 5.5) was utilized and scanning of the resolved bands at 215 nm. Linearity of the developed TLC method was evaluated and evident to be 0.4–2, 0.4–2.2, and 0.4–2 μg/band for MIC, NYS, and MET, in that order. The suggested chromatographic methods were verified according to ICH directives. The findings of the developed chromatographic procedures were statistically compared with the results of the reported ones using student’s t-test and F-test. Furthermore, two green assessment tools evaluated the indicated methods' level of greenness (GAPI and AGREE).

**Supplementary Information:**

The online version contains supplementary material available at 10.1186/s13065-023-01083-1.

## Introduction

Miconazole nitrate (MIC) belongs to the imidazole class of antifungal medications [1]. It functions by inhibiting the fungal growth that cause infectious diseases and employed topically to treat *Candida* infections like vaginitis [2]. Besides, Nystatin (NYS) is a polyene macrolide antifungal drug that is only used to treat superficial infectious *Candida*. NYS is prescribed to manage infections such as oral candidiasis and vaginitis [2]. Likewise, Metronidazole (MET) exerts antibacterial activity against anaerobes and several protozoa, and it inhibits *E. histolytica*, *G. lamblia* and *T. vaginalis* [2].

After conducting a review of the available literature, it was concluded that various analytical techniques for determining the three drugs under study as binary combinations had been described. Whereas, MIC and MET in their binary mixture were determined using HPLC [3–6], gas chromatography [5, 7], TLC-densitometric [7] and spectroscopic [8] methods. Similarly, MIC and NYS were determined as binary mixture by HPLC [9, 10], TLC [9] and spectrophotometric methods [10–12]. Besides, MET and NYS were determined by spectroscopic [13] and UV-chemometric methods [14].

Although there were various methods described for the determination of the cited drugs as binary mixtures, there was no method for separation of the three studied drugs as a ternary mixture. Accordingly, the present work introduces a smart chromatographic solution for the separation of the ternary mixture. However, MIC and NYS or MET and NYS are co-formulated as binary mixtures, we aimed in this work to develop and validate HPLC and TLC chromatographic methods for analyzing both mixtures (incorporate the three drugs) in one step, in the same run, and under the same circumstances. From the prospective of environmental impact, it is beneficial to operate a one-step analytical method instead of two methods for separating the same components. In agreement with the green analytical chemistry (GAC) principles [15], principle 4 which endorses the integration of analytical procedures and principle 8 which praises multianalyte determination than one analyte. Furthermore, principles 7 and 9 are attained by reducing waste-generation and saving energy-consumption via running single process rather than two processes.

The privileges of the presented HPLC and TLC methods are that they are fast, selective, time saving, sensitive, and do not require any special software. Hence, the developed chromatographic methods could be used for the separation of the studied compounds either found as ternary or binary mixtures in different pharmaceutical formulations with the same mobile phase in short analysis run time.

## Experimental

### Instruments

#### For HPLC method

HPLC device (Agilent 1260 infinity, Germany). Agilent 1260 infinity preparative ternary solvent delivery pump (G1361A) and auto sampler (G2260A) were used in this HPLC system. Agilent 1260 infinity thermostated column compartment (G1316A) and diode array detector (DAD). Separation and quantitation of the cited drugs were performed at room temperature on ZOBRAX Eclipse Plus RP- C_8_ column (25 cm × 4.6 mm i.d, 5 µm. USA). Sonix 4 ultrasonic (Sonix IV corporation, USA). Digital balance (Sartorius, Germany). 0.45 µm Nylon filter used to filter sample preparations.

#### For TLC method

Short wavelength 254 nm UV light lamp (Massachusetts, USA). CAMAG TLC scanner 3 S/N 130319 was used to scan samples bands which were controlled with winCATS program (Muttenz, Switzerland). Certain specifications are considered involving radiation source (Deuterium lamp), absorbance mode for scanning, chromatogram output (integrated peak area), slit dimension (3 × 0.45 mm), sample applicator (Linomat V auto sampler with 100 μL micro syringe), and scanning speed (20 mm/s) was used to apply the samples (CAMAG, Switzerland). TLC aluminum plates (20 × 20 cm) pre-coated with 0.25 mm silica gel 60 F254 (Merck, Germany).

### Chemicals and reagents

#### Samples

##### Standard drugs

Standard MET & NYS were graciously provided by EIPICO Egyptian Int. pharmaceutical Ind. Co. (10th of Ramadan city, Egypt). According to the reported method, their authenticity was 99.8% and 99.7%, respectively [6, 10]. Pharco Pharmaceuticals Industries (Alexandria, Egypt) generously afforded standard MIC with a purity of 99.7% in accordance with the reference method [1].

##### Pharmaceutical formulations

Monicure plus^®^ vaginal suppository (batch No. 7373004) was produced by Pharaonia pharmaceuticals (Alexandria, Egypt). Each suppository is declared to encompass 100 mg of MIC and 100,000 I.U equivalent to 20.5 mg of NYS [10, 16]. Amrizole N^®^ vaginal suppository (batch No. 977243) was manufactured by Amriya Pharmaceutical industry (Alexandria, Egypt). Each vaginal suppository of Amrizole N^®^ contains 100,000 I.U = 20.5 mg of NYS [10, 16] and 500 mg of MET.

#### Chemicals and solvents

All the following chemicals and reagents used to develop those methods were of analytical grade and used without further purification. Methanol HPLC grade (Sigma Aldrich Chemie GmbH, Germany). Sodium dodecyl sulfate, ethyl acetate, and toluene of analytical grade (El-Nasr pharmaceutical chemical Co., Abu- Zabaal, Cairo, Egypt). Deionized water (SEDICO pharmaceuticals Co., 6th October City, Egypt). Triethyl amine and formic acid (SDFCL, Mumbai, India).

### Standard solutions

#### Stock standard solution (1 mg/mL)

Standard stock solutions of MIC, NYS and MET were prepared using methanol at a concentration of 1 mg/mL. 100 mg of each drug were weighed and transferred into three separate 100-mL volumetric flasks (NYS should be prepared protected from light by its preparation in amber glass flask) then 50 mL of methanol was added. The prepared flasks were shaken to assure proper component dissolution. Methanol was then added to bring the volume up to 100 mL.

#### Working standard solution (0.1 mg/mL)

Standard MIC, NYS and MET working solutions were prepared at a concentration of 100 μg/mL. Three 100-mL volumetric flasks were accurately and independently filled with 10 mL of the set stock solutions of each component. The volume of the flask was completed using the mobile phase (methanol: 0.05% aqueous solution of sodium dodecyl sulfate (40: 60 v/v)) for HPLC method, while for TLC method methanol was used.

#### Preparation of laboratory mixtures

Different synthetic mixtures were produced using various MIC, NYS, and MET proportions. Into 10 mL measuring flask, different accurate volumes were transferred from their corresponding working solutions (0.1 mg/mL). The volume of the flask was completed using the mobile phase (methanol: 0.05% aqueous solution of sodium dodecyl sulfate (40: 60 v/v)) for HPLC method, while for TLC method methanol was used.

#### Preparation of pharmaceutical formulations

Five suppositories of Monicure plus^®^ and Amrizole N^®^ were weighed precisely and fine crashed separately in a mortar. From the crashed suppository, an amount equal to 0.025 mg of MET, MIC, and NYS were transferred individually into 25-mL volumentric flasks, 10 mL of methanol was added then subjected to a 30 min ultrasonic. The volumetric flasks of each pharmaceutical formulation were allowed to cool, and then solution was filtered. Following filtration, the residue was washed twice with 5 mL of methanol each time, and the volume was made up to produce a stock solution with a concentration of 1 mg/mL. By using the proper dilutions of the prepared stock solutions, working solutions for each pharmaceutical formulation at a concentration of (0.1 mg/mL) were produced. For the final dilution, methanol was used for the TLC method and mobile phase for the HPLC method.

### Procedure

#### Chromatographic conditions

##### For HPLC method

Chromatographic separation of MIC, MET and NYS was achieved by using ZOBRAX Eclipse Plus RP- C_8_ column (25 cm × 4.6 mm i.d, 5 µm. USA). Mixture of the three studied drugs was well resolved using isocratic elution of methanol: 0.05% aqueous solution of sodium dodecyl sulfate (40: 60 v/v). The mobile phase was filtered using a 0.45 m filter before use. The mobile phase’s flow rate was kept constant at 0.8 mL/min. The column temperature was monitored at 25 °C. Separated components were measured at a 220 nm wavelength. The injection had a 20 µL volume. The whole analysis run time was 6 min, and the total peak area was used to quantify the drugs under study.

##### For TLC method

By handling a Camag Linomat V applicator, the analysis was carried out on 20 × 10 cm TLC aluminum plates pre-coated with 0.25 mm silica gel 60 F_254_ and the investigated drugs were implemented to the TLC plates as bands of 4 mm width. The bands were employed 15 mm apart from the bottom edge of the plate and were spaced 5 mm from each other. The chromatographic chamber was pre-saturated for 30 min using the developing system. Plates were developed by ascending chromatography using ethyl acetate: toluene: methanol: triethylamine: formic acid (3: 1: 7: 0.3: 0.1 by volume) (pH = 5.5) as a developing solvent at a distance of 8 cm. At room temperature, the plates were left to dry in air and the bands of the separated components were detected under UV lamp at 215 nm.

#### Construction of calibration curves

##### For HPLC method

Separately and precisely, aliquots of MIC, NYS, and MET from their respective working standard solutions (0.1 mg/mL) were transferred into three series of 10-mL volumetric flasks, each corresponding to 50–500, 40–500, and 40–400 µg, respectively. Mobile phase was used as a diluent. For construction of the calibration curve, peak areas of the recorded chromatograms were used, relating those peak areas to the corresponding concentration of each component. For each drug, regression equation was calculated from the previously constructed calibration graphs.

##### For TLC method

Separately and precisely, aliquots corresponding to 0.4–2.00, 0.4–2.20, and 0.4–2.00 mg of MIC, NYS, and MET were transmitted into three series of 10-mL volumetric flasks from their respective stock standard solutions (1 mg/mL). To fill the volume of the prepared flasks, methanol was used. 10 µL of each solution were employed in triplicate on the TLC plates in accordance with the instructions outlined under the chromatographic condition. The wavelength used for scanning the plates was 215 nm and the areas under the peaks were recorded. To create the calibration curve for each cited drug, the integrated peak area (× 10^–4^) was correlated to the associated concentration of each component. Then, the linear regression equation for each drug was calculated from their constructed calibration curves.

#### Application to pharmaceutical formulations

The procedures mentioned under preparation of pharmaceutical formulations were adopted to extract MIC, NYS and MET from their pharmaceutical preparations. The concentrations of MIC, NYS, and MET were measured from their relevant regression models using the method described under the construction of calibration curves. Different known concentrations of pure MIC and NYS were spiked to pre-analyzed Monicure plus^®^ vaginal suppositories for the purpose of evaluating the method's accuracy. NYS and MET were spiked to pre-analyzed Amrizole N^®^ vaginal suppositories. Following that, the steps outlined under “construction of calibration curve” were taken. Afterward, concentrations of the three cited drugs were calculated and the average recovery percent was determined for each pure added drug.

## Results and discussion

Because of its outstanding resolution and wide range of stationary and mobile phases, which allow for the study of several compounds with various polarity, chromatographic techniques are the ideal method for distinguishing and determining multianalyte mixtures of components [17–20].

Liquid chromatography has been used to separate mixture of components using either columns as in HPLC [21, 22] or plates as in TLC [23, 24]. Several methods for determining the cited drugs were reported for their measurement in their binary mixtures or with other components, nonetheless no current technique adopted for separation of the three drugs as a ternary mixture.

From the environmental and economic standpoints, it is favored to run one method instead of two methods to determine the same components. Additionally, from the analytical point of view, establishing single method for multianalytes separation will save time and energy, reduce waste, and keep workers safe. Herein, fully developed, validated, and optimized HPLC and TLC chromatographic methodologies were exploited for separation of MIC, NYS, and MET in single run within short analysis time simultaneously.

### Optimization of the analytical method

The influence of several parameters that can have a significant impact on the selectivity, efficacy, and sensitivity of the chromatographic resolution was tested to improve the recommended HPLC and TLC procedures. These variables include the type, polarity, and ratios of the mobile phase, various scanning wavelengths (for HPLC and TLC procedures), flow rate of the mobile phase (for HPLC), and band diameters that were improved (for TLC method).

#### For HPLC method

The components under examination have various physicochemical characteristics, which makes their chromatographic separation challenging. MET has pK_a_ = 2.62, whereas NYS pK_a_ = 3.62, and MIC pK_a_ = 6.77 [25]. Besides, MET is a hydrophilic drug with log K_ow_ =− 0.1, but NYS retains log K_ow_ = 7.08, and MIC owns log K_ow_ = 5.96 [26].

**Fig. 1:**
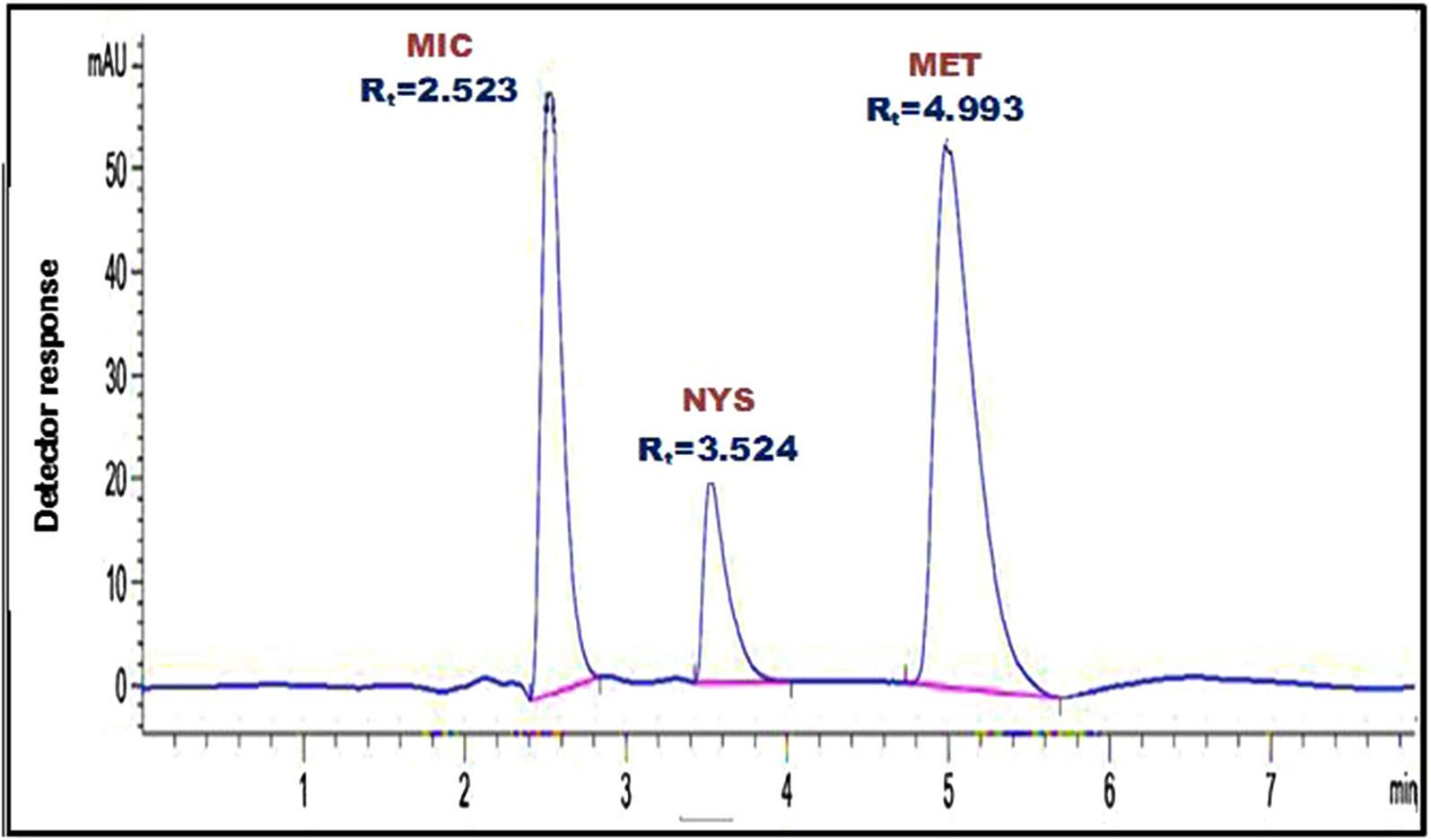
RP-HPLC chromatogram of the separated mixture of MIC, NYS, and MET using methanol: 0.05% aqueous solution of sodium dodecyl sulfate (40:60, v/v) as the mobile phase at 220 nm

Different compositions, ratios and polarities of mobile phase were investigated to separate the ternary mixture. Firstly, for separation of the three cited components methanol was used in different ratio with pure water. The first run was performed using methanol: water in equal ratio (50: 50 v/v). Upon using this mobile phase MIC has a forked peak and not resolved from NYS. Whilst MET was separated with slight tailing. By increasing the ratio of methanol, MET has symmetric peak, but MIC forked peak became more obvious and eluted at the same retention time as NYS. The effect of addition of glacial acetic acid or triethylamine in different ratios was tested but it gave bad result regarding the peak resolution and symmetry. Many trials were performed to achieve more sharp and symmetric peaks with high resolution and better sensitivity. The best result was obtained upon using sodium dodecyl sulfate (SDS) in concentration (0.05%) with methanol. Using SDS was found to have a great effect on peak symmetry and resolution for the three drugs. This might be due to micelle formation which allow separation of components of different polarities [27]. MET has a sharper and more symmetric peak. MIC and NYS were well resolved with sharp and more symmetric peaks in short analysis time (6 min). Complete and best separation was achieved using methanol: 0.05% aqueous solution of sodium dodecyl sulfate (40: 60 v/v). MIC, NYS, and MET were completely resolved at retention time of 2.523, 3.524 and 4.993 min, respectively, as illustrated in Fig. 1.

To properly identify the wavelength with the highest sensitivity for the detection of the separated compounds, different scanning wavelengths (215, 220, and 230 nm) were explored. The best wavelength used for detection of the cited drugs was found to be 220 nm regarding sensitivity with minimum noise detected, as demonstrated in Fig. 1.

Also, it was attempted to see how the flow rate of the mobile phase affected the chromatographic separation. Flow rates for the mobile phase delivery ranged from 0.5 to 1.5 mL/min; a flow rate 0.8 mL/min was evident to give maximum separation and best resolution with no interference between the peaks and minimum analysis time (6 min). Individual chromatogram for the studied components showing their T_R_ is revealed in Additional file 1: Fig. S1.

#### For TLC method

For separation of the three studied compounds with high resolution using TLC plates, different developing systems varied in ratios and polarities were inspected e.g. (methanol: chloroform) and (methanol: chloroform: glacial acetic acid). All the previously mentioned developing systems failed to separate the studied drugs with good resolution as MIC and MET weren’t resolved well from each other while NYS was retained on the baseline which revealed high polarity. Adding water for the previous developing system allows NYS to move from baseline but MIC and MET weren’t resolved and run on the solvent front. Upon replacement of chloroform with ethyl acetate which has slightly higher polarity, NYS started to move from baseline with tailing but MIC and MET still not resolved very well. Accordingly, toluene was added in small proportion (miscibility of the developing system was checked) enabling better resolution of MIC and MET. Maximum resolution between the MIC and MET was achieved upon using triethyl amine which also have a significant effect on NYS which migrate from the baseline with slight tailing, this tailing was controlled by using formic acid where the spots were more compact for the three cited drugs.

**Fig. 2:**
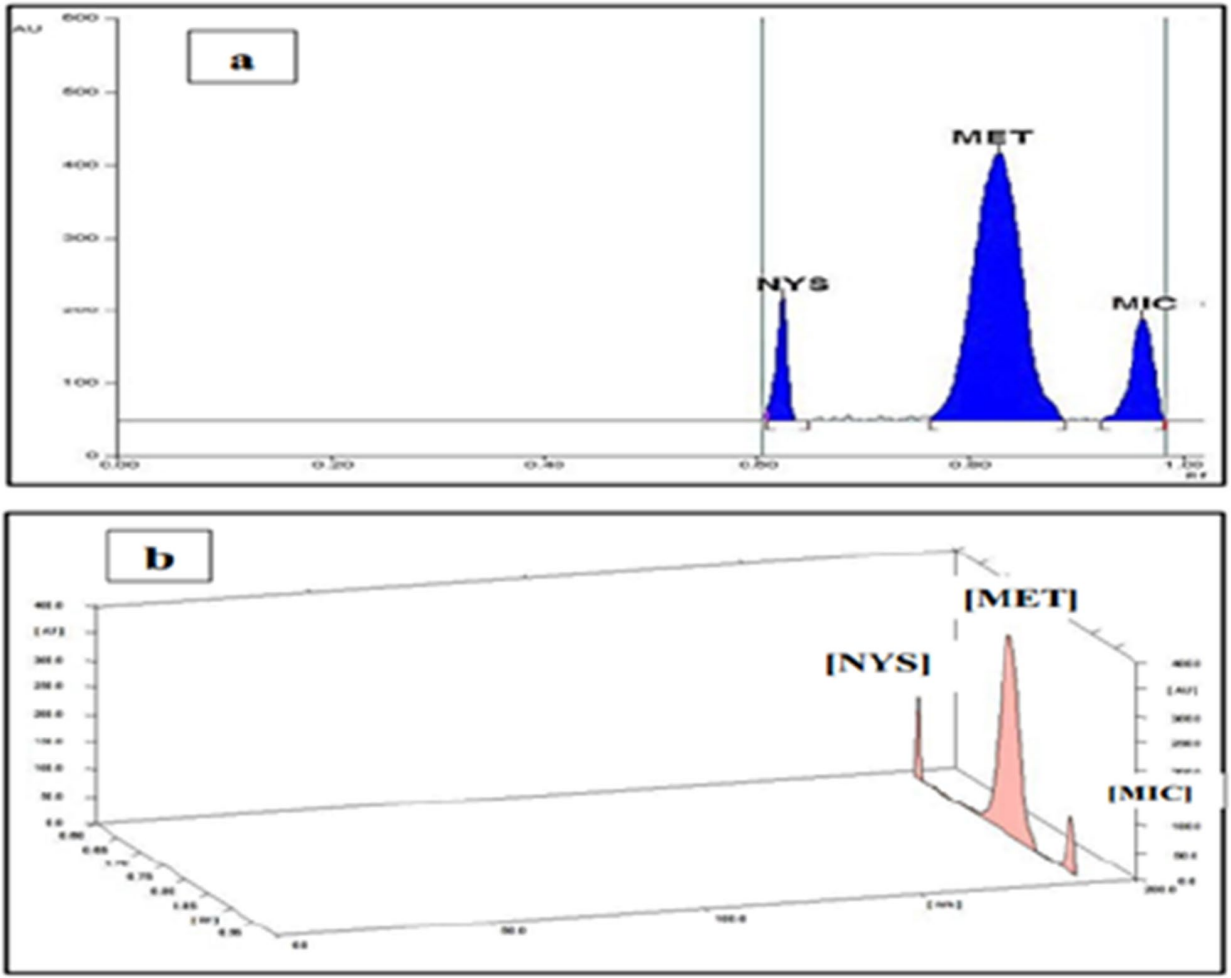
**a** 2D and **b** 3D HPTLC chromatogram of resolved mixture of standard NYS (R_f_ = 0.62), MET (R_f_ = 0.83) and MIC (R_f_ = 0.96) by using ethyl acetate: toluene: methanol: triethylamine: formic acid (3:1:7:0.3:0.1, by volume) (pH = 5.5) as a developing system and scanning at 215 nm

Complete separation of the ternary mixture with high resolution and sharp and symmetric peaks was accomplished upon using a developing solvent comprises ethyl acetate: toluene: methanol: triethyl amine: formic acid (3: 1: 7: 0.3: 0.1 by volume) (at pH = 5.5) where the difference in retention factor were found to be (R_f_) for (NYS = 0.62), (MET = 0.83) and (MIC = 0.96), as shown in Fig. 2.

Several scanning wavelengths (215, 220, 230, and 254 nm) were verified, however it was observed that scanning at 215 nm exhibited well-separated, sharp, and symmetric peaks with the least amount of background noise and the highest sensitivity for the three drugs specified, as displayed in Fig. 2.

Besides, to obtain sharp and symmetric separated peaks, different band dimensions were studied. Taking into consideration the range of concentration applied and number of tracks applied on the plates, the optimal dimensions were band with 4 mm width and 5 mm inter-space. likewise, various slit dimensions were assessed, and the best slit dimension was found to be (3 mm × 0.45 mm) which provided the highest sensitivity for the studied compounds, recognizing that the scanning light beam slit dimension should cover the dimensions of the band on the scanned track without interference from any adjacent bands. Individual chromatogram for the separated components showing their R_f_ is disclosed in Additional file 1: Fig. S2.

### Validation of the analytical method

The suggested methodologies were approved in accordance with ICH requirements [28]. Validation parameters including linearity, LOD, LOQ and precision were determined, and results were listed in Table 1.

**Table 1:** Regression and validation parameters of the proposed HPLC and HPTLC methods for determination of MIC, NYS, and MET

Parameters	HPLC			HPTLC		
	MIC	NYS	MET	MIC	NYS	MET
Linearity	5–50 μg/mL	4–50 μg/mL	4–40 μg/mL	0.4–2 μg/band	0.4–2.2 μg/band	0.4–2 μg/band
Slope	13.9680	4.3515	28.2340	0.4762	0.8460	1.102
Intercept	− 0.9486	193.2800	3.8488	0.2186	− 0.2224	0.4583
Correlation coefficient	0.9999	0.9999	0.9999	0.9999	0.9998	0.9999
Accuracy (%)	100.41	100.42	99.19	99.53	100.28	100.00
Repeatability (RSD%)^a^	0.17	0.62	0.61	0.80	0.49	0.77
Intermediate Precision (RSD%)^b^	0.71	0.82	0.66	1.33	0.67	1.69
LOD^c^	1.19 μg/mL	0.95 μg/mL	0.80 μg/mL	0.11 μg/band	0.09 μg/band	0.07 μg/band
LOQ^c^	3.62 μg/mL	2.87 μg/mL	2.43 μg/mL	0.34 μg/band	0.27 μg/band	0.22 μg/band

^a^The intraday precision (n = 3), average of three different concentrations repeated three times within day

^b^The interday precision (n = 3), average of three different concentrations repeated three times in three successive days

^c^Limit of detection and quantitation are determined via calculations: LOD = (SD of the response/slope) × 3.3; LOQ = (SD of the response/slope) × 10

The linearity of the two established methods was evaluated under the chromatographic circumstances previously described, and it was found to be in the concentration range of 5–50, 4–50 and 4–40 µg/mL for MIC, NYS, and MET, respectively, for HPLC method. Linearity for TLC method was evaluated and found to be 0.4–2, 0.4–2.2 and 0.4–2 μg/band for MIC, NYS, and MET, respectively.

Samples of different concentrations of pure drugs within their concentration ranges were analyzed to verify the accuracy of the suggested chromatographic methods. Good percentage recoveries were obtained indicating good accuracy of the two developed methods, Additional file 1: Table S1. The stability of prepared solutions for either individual drugs or their ternary mixtures were checked by injection in HPLC or application to TLC at different time interval for 24 h. whereas, the obtained chromatograms indicate no change between peaks of individual drugs and that obtained from ternary mixture combination revealing that there is no interaction between the studied drugs.

Applicability and validity of the developed method proved through analysis of the three cited compounds in their pharmaceutical formulations. Standard addition technique was then applied for further assessment of validity and accuracy of the two newly developed methods. Good and accurate results indicate no interference from the encountered excipients found in their pharmaceutical formulations, as explained in Table 2.

**Table 2:** Quantitative determination of NYS and MET in Amrizole N^®^ vaginal suppository and NYS and MIC in Monicure plus^®^ vaginal suppository by the proposed HPLC and HPTLC methods and application of standard addition technique

Pharmaceutical formulation	Drug	HPLC				HPTLC			
		Taken (μg/mL)	Found^a^ % ± SD	Pure added (μg/mL)	%Recovery^b^	Taken (μg/band)	Found^a^ % ± SD	Pure added (μg/band)	%Recovery^b^
Amrizole N® vaginal suppository Batch NO. (977,243)	NYS	40.00	0.57 ± 99.08	3.00	99.27	1.00	0.86 ± 99.11	0.60	100.00
				6.00	98.42			0.80	101.11
				9.00	98.96			1.00	99.60
		SD ± Mean		98.88 ± 0.43		SD ± Mean		0.78 ± 100.24	
	MET	12.00	± 102.55 0.44	6.00	98.39	1.00	± 102.32 0.27	0.50	99.94
				12.00	98.31			0.60	100.50
				18.00	98.52			1.00	98.96
		SD ± Mean		0.11 ± 98.41		SD ± Mean		0.78 ± 99.80	
Monicure plus® vaginal suppository Batch NO. (7,373,004)	NYS	40.00	± 101.28 0.66	3.00	100.87	1.00	± 101.03 0.44	0.60	100.00
				6.00	100.13			0.80	100.38
				9.00	100.40			1.00	100.16
		SD ± Mean		0.38 ± 100.47		SD ± Mean		0.19 ± 100.18	
	MIC	15.00	± 102.28 0.33	10.00	100.16	1.00	± 102.03 0.54	0.60	98.81
				15.00	99.37			0.80	98.16
				25.00	98.45			1.00	98.36
		SD ± Mean		0.86 ± 99.33		SD ± Mean		0.33 ± 98.44	

^a^Average of six experiments

^b^Average of three experiments

The method robustness for determination of MIC, NYS, and MET was also evaluated during method validation to determine how system suitability parameters, e.g. (the symmetry factor (T), column capacity (K′), resolution (Rs) and recovery percent), would be affected by minor deliberate variations in experimental conditions. Robustness for HPLC method was judged by changing the ratio of methanol in the mobile phase (± 5%) and flow rate (± 0.2.) No considerable changes were observed in the studied parameters, as listed in Additional file 1: Table S2. Whereas robustness for TLC method was evaluated by changing the ratio of toluene in the developing system (± 0.1%), triethylamine (± 0.05) and the wavelength (± 2 nm). No remarkable variations were observed in the studied parameters, as indicated in Additional file 1: Table S2.

To confirm the applicability and reproducibility of liquid chromatographic method for analysis, tests were conducted to verify suitability of the system by determining various parameters [29] e.g. selectivity, capacity factor, resolution and others. Values of system suitability parameters were calculated according to USP [29], and the results were found to be in the accepted ranges, as summarized in Table 3.

**Table 3:**
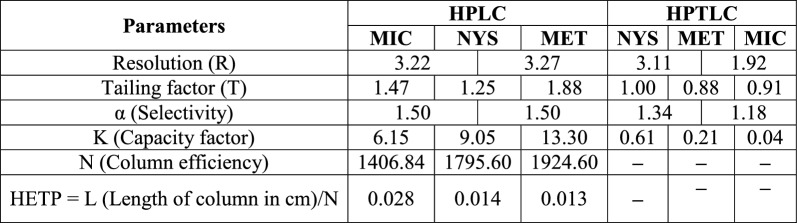
System suitability testing parameters of the developed HPLC and HPTLC methods

Using student's t-test and F-test statistics, findings from the two constructed HPLC and TLC procedures and the described methods [6, 10] were compared. Between the two proposed procedures and the published methodologies, there was no meaningful variation in the reliability and exactness as evidenced by the estimated values of t- and F- being lower than the tabulated ones, Table 4.

**Table 4:** Statistical comparison of the results obtained by the proposed methods and the reported method for determination of MIC, MET and NYS in their pharmaceutical formulation

Parameters	HPLC method		HPTLC method		Reported method [6]	
	MIC	NYS	MIC	NYS	MIC	NYS
Monicure plus^®^ vaginal suppository						
Mean %	102.28	101.28	102.03	101.03	102.37	101.01
SD	0.33	0.66	0.54	0.44	0.43	0.65
n	6	6	6	6	6	6
Student’s *t*-test (2.228)^a^	0.410	0.710	1.210	0.080	–	–
*F*-value (5.050)^a^	1.720	1.010	1.560	2.220	–	–

Parameters	HPLC method		HPTLC method		Reported method [10]	
	MET	NYS	MET	NYS	MET	NYS
Amrizole N^®^ vaginal suppository						
Mean %	102.01	99.08	102.32	99.11	102.39	99.08
SD	0.62	0.57	0.27	0.86	0.31	0.59
n	6	6	6	6	6	6
Student’s *t*-test (2.228)^a^	1.380	0.002	0.430	0.070	–	–
*F*-value (5.050)^a^	3.940	1.060	1.290	2.130	–	–

^a^Figures in parentheses represent the corresponding tabulated of *t* and *F* at P = 0.05

## Greenness profile assessment

To address the ecological impact of the developed chromatographic methods, two greenness assessment tools were appointed termed the Green Analytical Procedure Index (GAPI) [30] and AGREE [31].

GAPI is a three-colored symbol that has a design made up of 15 pictograms, each of which stands for a stage in the analytical process. Every analytical step, including sample preparation and final analysis, is evaluated, and categorized into three sections: red, yellow, and green. These classifications correlate to environmental effect levels of low, moderate, or high. Analyst could identify and judge the environmental effects of established analytical procedures using the GAPI approach, enabling the adoption of greener practices [32, 33]. For the proposed HPLC method, out of all pictograms 5 are green, 7 yellow, and only 3 red, whilst TLC method acquired 4 green sections, 7 yellow and only 4 shaded red. However, the reported methods for determination of MIC/MET [6] and MIC/NYS [10] acquired less green and more yellow and red shades, which indicated that the proposed chromatographic procedures are environmentally benign and greener than the reported ones, Table 5.

**Table 5:** Pictograms for Greenness assessment of the developed HPLC and TLC methods using GAPI and AGREE tools compared to the reported methods

Greenness profile	Proposed methods		Reported methods	
	HPLC	TLC	MIC/MET mixture [6]	MIC/NYS mixture [10]
GAPI				
AGREE				

The 12 green chemistry concepts were used to build the AGREE software. Each concept is rated between green to red (1.0 to 0.0) relying on its score [31]. A clock-shaped graph reflects the outcome, with one color in the center representing the inclusive score. The quantitative and qualitative output diagrams allow an accurate assessment of the procedure's greenness. Also, the AGREE tool highlights both the advantages and disadvantages of each analytical method, so one can determine their relative importance in each sector according to their strengths and weaknesses [34].

Based on the AGREE assessment, TLC and HPLC processes were both rated as green approaches with 0.58 overall scores. However, the reported methods received 0.47 and 0.51 ratings. These reported methods demonstrated a low green score because they use many solvents that are not green (acetonitrile) and run under long analysis time (up to 22 min per run) that consume large amounts of solvent. Table 5 clearly illustrates that the suggested approaches outperform the published chromatographic methods used to analyze drugs under investigation in terms of GAPI and AGREE pictograms.

## Conclusion

From the prospective of GAC, Herein two green chromatographic techniques were designed to miniaturize solvent-consumed, energy-utilized, waste-created, and analysis-time by exploiting single-step HPLC and TLC methods. For the detection of MIC, NYS, and MET encountered together as ternary mixtures, two distinctive, sensitive, and selective HPLC and TLC approaches were created, optimized, and validated. TLC method has the privilege of being time saving and low cost per analysis as it allows analysis of several samples simultaneously by using small amount of developing system with high sensitivity and selectivity. The main advantages of HPLC-DAD method are being rapid and selective. It is able to resolve the three studied substances in a brief analysis period (6 min), reducing the amount of solvent utilized. Accordingly, the developed method is economically efficient and time saving. The two developed chromatographic methods were used for analysis of the three cited drugs in two different dosage forms combined as binary mixture by using one mobile phase for all dosage forms with high accuracy and precision. In addition, GAPI and AGREE assessment tools were used to determine the environmental friendliness of the methods presented. Hence, the proposed methodologies might be applied for the analysis of MIC, NYS, and MET whether in their pure state or in their drug products.

### Supplementary Information

**Additional file 1:** **Table S1.** Accuracy results as % recovery for determination of MIC, NYS and MET by the proposed HPLC and HPTLC methods. **Table S2.** Experimental results of robustness for determination of MIC, NYS and MET by the developed HPLC and HPTLC methods. **Figure S1.** HPLC chromatograms for individual peaks of MET, NYS, and MIC. **Figure S2.** TLC chromatograms for calibration curves for MET, NYS, and MIC.

## Data Availability

All data generated or analyzed during this study are included in this published article and its supplementary information files.

## References

[1] British Pharmacopoeia, Stationary Office, Appendix 1D, Buckingham Palace Road, London, UK; 2016.

[2] Craig CR, Stitzel RE (2004). Modern pharmacology with clinical applications.

[3] Akay C, Özkan SA, Şentürk Z, Cevheroğlu Ş (2002). Simultaneous determination of metronidazole and miconazole in pharmaceutical dosage forms by RP-HPLC. Farm.

[4] Aldewachi HS, Gurram A (2013). A comparative study of two chromatographic techniques for the determination of group of imidazoles simultaneously. Int J Pharm Sci Rev Res.

[5] Ashour S, Kattan N (2010). Simultaneous determination of miconazole nitrate and metronidazole in different pharmaceutical dosage forms by gas chromatography and flame ionization detector ( GC-FID ). Int J Biomed Sci.

[6] Sahoo DR, Jain S (2016). A rapid and validated RP-HPLC method for the simultaneous quantification of benzoic acid, metronidazole and miconazole nitrate in vaginal formulations. J Chromatogr Sci.

[7] Meshram DB, Bagade SB, Tajne MR (2009). Simultaneous determination of metronidazole and miconazole nitrate in gel by HPTLC. Pak J Pharm Sci.

[8] Erk N, Levent Altun M (2001). Spectrophotometric resolution of metronidazole and miconazole nitrate in ovules using ratio spectra derivative spectrophotometry and RP-LC. J Pharm Biomed Anal.

[9] Salama I, Gomaa MS (2013). Comparative determination of miconazole, nystatin, hydrocortisone and neomycin by HPTLC/HPLC-DAD. Eur J Chem.

[10] Heneedak HM, Salama I, Mostafa S, El-sadek M (2012). HPLC and chemometric methods for the simultaneous determination of miconazole nitrate and nystatin. J Chromatogr Sci.

[11] Sayed RA, Mohamed AR, Hassan WS, Elmasry MS (2021). Smart UV-spectrophotometric platforms for rapid green analysis of miconazole nitrate and nystatin in their combined suppositories and in vitro dissolution testing. Drug Dev Ind Pharm.

[12] Mohamed AR, Abolmagd E, Badrawy M, Nour IM (2022). Innovative earth-friendly UV-spectrophotometric technique for in vitro dissolution testing of miconazole nitrate and nystatin in their vaginal suppositories: greenness assessment. J AOAC Int.

[13] Korany MA, Abdine HH, Ragab MAA, Aboras SI (2015). Application of derivative spectrophotometry under orthogonal polynomial at unequal intervals: determination of metronidazole and nystatin in their pharmaceutical mixture. Spectrochim Acta Part A Mol Biomol Spectrosc.

[14] Baratieri SC, Barbosa JM, Freitas MP, Martins JA (2006). Multivariate analysis of nystatin and metronidazole in a semi-solid matrix by means of diffuse reflectance NIR spectroscopy and PLS regression. J Pharm Biomed Anal.

[15] Gałuszka A, Migaszewski Z, Namieśnik J (2013). The 12 principles of green analytical chemistry and the SIGNIFICANCE mnemonic of green analytical practices. TrAC Trends Anal Chem.

[16] Lightbown JW, Kogut M, Uemura K (1963). The international standard for nystatin. Bull World Health Organ.

[17] Abd El-Hadi HR, Eltanany BM, Zaazaa HE, Eissa MS (2023). Chromatographic impurity profile assessment of dual action binary combination: ecological analysis with comparative statistics”. Sustain. Chem. Pharm..

[18] Magdy MA, Farid NF, Anwar BH, Abdelhamid NS (2023). A validated ecofriendly chromatographic method for determination of myasthenia gravis combined medications in spiked human plasma. Biomed Chromatogr.

[19] Gamal M, Naguib IA, Abdelfatah RM (2021). Simultaneous analysis of oxytetracycline hydrochloride, lidocaine, and bromhexine hydrochloride in the presence of many interfering excipients. Arch Pharm (Weinheim).

[20] Ali HM, Gamal M, Ghoneim MM, Mohammed Abd Elhalim L (2022). Quantitative analysis of abamectin, albendazole, levamisole HCl and closantel in Q-DRENCH oral suspension using a stability-indicating HPLC-DAD method”. Molecules.

[21] Fares MY, Abdelwahab NS, Abdelrahman MM, Abdel-Rahman HM (2019). Determination of sofosbuvir with two co-administered drugs; paracetamol and DL-methionine by two chromatographic methods. Application to a pharmacokinetic study. Bioanalysis.

[22] Abdelwahab NS, Abdelrahman MM, Boshra JM, Taha AA (2019). Different stability-indicating chromatographic methods for specific determination of paracetamol, dantrolene sodium, their toxic impurities and degradation products”. Biomed Chromatogr.

[23] Habib NM, Abdelrahman MM, Abdelwhab NS, Ali NW (2017). Validated chromatographic methods for the analysis of two binary mixtures containing pyridoxine hydrochloride. J AOAC Int.

[24] Abbas SS, Wagieh NE, Abdelkawy M, Abdelrahman MM (2011). Simultaneous determination of diloxanide furoate and metronidazole in presence of diloxanide furoate degradation products. J AOAC Int.

[25] Babić S, Horvat AJM, Mutavdžić Pavlović D, Kaštelan-Macan M (2007). Determination of pKa values of active pharmaceutical ingredients. TrAC Trends Anal Chem.

[26] Hsieh C-M, Wang S, Lin S-T, Sandler SI (2011). A predictive model for the solubility and octanol−water partition coefficient of pharmaceuticals. J Chem Eng Data.

[27] Fuguet E, Ràfols C, Rosés M, Bosch E (2005). Critical micelle concentration of surfactants in aqueous buffered and unbuffered systems. Anal Chim Acta.

[28] ICH (2005). Validation of an analytical procedures: text and methodology Q2(R1), guidance, November 1996.

[29] Brown W, Marques MR, Shargel L, Kanfer I (2013). 14 The United States Pharmacopeia/National Formulary. Generic drug product development: solid oral dosage forms.

[30] Płotka-Wasylka J (2018). A new tool for the evaluation of the analytical procedure: green analytical procedure index. Talanta.

[31] Pena-Pereira F, Wojnowski W, Tobiszewski M (2020). AGREE—analytical GREEnness metric approach and software. Anal Chem.

[32] Imam MS, Abdelrahman MM (2023). How environmentally friendly is the analytical process? A paradigm overview of ten greenness assessment metric approaches for analytical methods. Trends Environ Anal Chem.

[33] Mahgoub SM, Mahmoud MR, Binsaleh AY, Almalki MA, Mohamed MA, Nassar HF (2023). Analytical assessment of a novel RP-HPLC method for the concurrent quantification of selected pharmaceutical drugs levodopa and carbidopa using eight greenness metrics comparing to the lean six sigma approach. Sustain Chem Pharm.

[34] Gamil M, El Zahar NM, Magdy N, El-Kosasy AM (2023). Greenness and whiteness evaluated triple divisor-continuous wavelet transform spectrophotometric and advanced chemometric methods for the simultaneous determination of four photoinitiators in saliva and intravenous injection solution. Sustain Chem Pharm.

