# Supplementary material for: Ecofriendly single-step HPLC and TLC methods for concurrent analysis of ternary antifungal mixture in their pharmaceutical products
Source: BMC Chem. 2023 Dec 1;17(1):173. doi: 10.1186/s13065-023-01083-1 (PMC10691124; doi:10.1186/s13065-023-01083-1)

**Additional file**

**Ecofriendly Single-Step HPLC and TLC Methods for Concurrent Analysis of Ternary Antifungal Mixture in Their Pharmaceutical Products**

Maha M. Abdelrahman ^a^, Ibrahim A. Naguib ^b^, Hala E. Zaazaa^c^, Hend M. Nagieb^d^

^a^ Pharmaceutical Analytical Chemistry Department, Faculty of Pharmacy, Beni-Suef University, Alshaheed Shehata Ahmad Hegazy St, 62514 Beni-Suef, Egypt.

^b^ Department of Pharmaceutical Chemistry, College of Pharmacy, Taif University, P.O. Box 11099, Taif 21944, Saudi Arabia.

^c^ Pharmaceutical Analytical Chemistry Department, Faculty of Pharmacy, Cairo University, Kasr El-Aini St., 11562, Cairo, Egypt.

^d^ Pharmaceutical Chemistry Department, Faculty of Pharmacy, Nahda University, Beni-Suef, Egypt.

Table S1: Accuracy results as % recovery for determination of MIC, NYS and MET by the proposed HPLC and HPTLC methods.

| Drug | HPLC | | | HPTLC | | |
| --- | --- | --- | --- | --- | --- | --- |
|  | Expected concentration (µg/mL) | Found concentration(µg/mL) | %  Recovery * | Amount spotted (µg/band) | Found concentration (µg/band) | %  Recovery * |
| MIC | 15.00 | 15.09 | 100.60 | 0.40 | 0.401 | 100.25 |
|  |  | 15.07 | 100.47 |  | 0.400 | 100.00 |
|  |  | 15.00 | 100.00 |  | 0.390 | 97.50 |
|  | 20.00 | 20.01 | 100.05 | 0.80 | 0.790 | 98.75 |
|  |  | 20.15 | 100.75 |  | 0.800 | 100.00 |
|  |  | 20.01 | 100.05 |  | 0.802 | 100.25 |
|  | 30.00 | 30.18 | 100.60 | 1.00 | 1.000 | 100.00 |
|  |  | 29.98 | 99.93 |  | 1.010 | 101.00 |
|  |  | 30.38 | 101.27 |  | 0.980 | 98.00 |
|  | Mean ±%RSD | 100.41 ± 0.44 | | Mean ±%RSD | 99.53 ± 1.17 | |
| NYS | 10.00 | 10.02 | 100.20 | 0.40 | 0.402 | 100.50 |
|  |  | 10.06 | 100.60 |  | 0.400 | 100.00 |
|  |  | 9.99 | 99.90 |  | 0.390 | 97.50 |
|  | 20.00 | 19.95 | 99.75 | 0.80 | 0.810 | 101.25 |
|  |  | 20.18 | 100.90 |  | 0.800 | 100.00 |
|  |  | 20.09 | 100.45 |  | 0.810 | 101.25 |
|  | 30.00 | 30.03 | 100.10 | 1.00 | 1.010 | 101.00 |
|  |  | 30.27 | 100.90 |  | 1.010 | 101.00 |
|  |  | 30.29 | 100.97 |  | 1.00 | 100.00 |
|  | Mean ±%RSD | 100.42 ± 0.46 | | Mean ±%RSD | 100.28 ± 1.17 | |
| MET | 12.00 | 11.94 | 99.50 | 0.60 | 0.590 | 98.33 |
|  |  | 11.96 | 99.67 |  | 0.600 | 100.00 |
|  |  | 11.97 | 99.75 |  | 0.610 | 101.67 |
|  | 24.00 | 23.54 | 98.08 | 1.00 | 0.990 | 99.00 |
|  |  | 23.62 | 98.42 |  | 1.000 | 100.00 |
|  |  | 23.71 | 98.79 |  | 1.010 | 101.00 |
|  | 30.00 | 29.76 | 99.20 | 1.40 | 1.410 | 101.71 |
|  |  | 29.91 | 99.70 |  | 1.390 | 99.29 |
|  |  | 29.87 | 99.57 |  | 1.400 | 100.00 |
|  | Mean ±%RSD | 99.19 ± 0.72 | | Mean ±%RSD | 100.00 ± 1.04 | |

*** Average of three determinations.**

Table S2: Experimental results of robustness for determination of MIC, NYS and MET by the developed HPLC and HPTLC methods.

| Drug | Robustness parameters | | | T^a^ | K'^a^ | Rs^b^ | % Assay ^c^ |
| --- | --- | --- | --- | --- | --- | --- | --- |
| HPLC | | | | | | | |
| MIC | Flow rate (mL/min) | 0.8 + 0.2 | | 1.50 | 6.30 | 3.20 | 99.62 |
|  |  | 0.8 - 0.2 | | 1.51 | 6.25 | 3.16 | 100.09 |
|  | % RSD | | | 0.47 | 0.56 | 0.89 | 0.33 |
|  | Methanol composition | 40% + 5% | | 1.47 | 6.27 | 2.96 | 99.97 |
|  |  | 40% - 5% | | 1.49 | 6.11 | 3.03 | 100.39 |
|  | % RSD | | | 0.96 | 1.83 | 1.65 | 0.30 |
| NYS | Flow rate (mL/min) | 0.8 + 0.2 | | 1.46 | 9.24 | 3.08 | 99.89 |
|  |  | 0.8 - 0.2 | | 1.41 | 9.00 | 2.98 | 100.52 |
|  | % RSD | | | 2.46 | 1.86 | 2.33 | 0.454 |
|  | Methanol composition | 40% + 5% | | 1.32 | 9.21 | 2.95 | 100.07 |
|  |  | 40% - 5% | | 1.30 | 8.96 | 3.02 | 100.21 |
|  | % RSD | | | 1.08 | 1.95 | 1.66 | 0.10 |
| MET | Flow rate (mL/min) | 0.8 + 0.2 | | 1.90 | 13.59 | ------ | 100.79 |
|  |  | 0.8 - 0.2 | | 1.85 | 13.44 | ------ | 101.07 |
|  | % RSD | | | 1.89 | 0.79 | ------- | 0.20 |
|  | Methanol composition | 40 + 5% | | 1.87 | 13.52 | ------ | 101.49 |
|  |  | 40 - 5% | | 1.89 | 12.85 | ------ | 101.15 |
|  | % RSD | | | 0.75 | 2.01 | ------- | 0.24 |
| HPTLC | | | | | | | |
| NYS | Toluene (mL) | | 1 + 0.1 | 1.01 | 0.68 | 3.04 | 99.46 |
|  |  |  | 1 - 0.1 | 0.98 | 0.65 | 2.98 | 99.36 |
|  | % RSD | | | 2.13 | 3.19 | 1.41 | 0.07 |
|  | Triethylamine (mL) | | 0.3 + 0.05 | 1.11 | 0.66 | 2.97 | 99.97 |
|  |  |  | 0.3 - 0.05 | 1.12 | 0.65 | 3.08 | 99.53 |
|  | % RSD | | | 0.63 | 1.08 | 2.57 | 0.31 |
|  | Scanning wavelength (nm) | | 215 + 2 nm | 1.00 | 0.57 | 3.05 | 99.09 |
|  |  |  | 215 - 2 nm | 1.02 | 0.56 | 3.10 | 99.19 |
|  | % RSD | | | 1.40 | 1.25 | 1.15 | 0.07 |
| MET | Toluene (mL) | | 1 + 0.1 | 0.87 | 0.27 | 1.87 | 99.28 |
|  |  |  | 1 - 0.1 | 0.90 | 0.28 | 1.83 | 98.98 |
|  | % RSD | | | 2.09 | 2.57 | 1.53 | 0.21 |
|  | Triethylamine (mL) | | 0.3 + 0.05 | 0.86 | 0.20 | 1.77 | 98.59 |
|  |  |  | 0.3 - 0.05 | 0.88 | 0.21 | 1.81 | 98.43 |
|  | % RSD | | | 1.63 | 2.40 | 1.58 | 0.12 |
|  | Scanning wavelength (nm) | | 215 + 2 nm | 0.87 | 0.21 | 1.62 | 99.32 |
|  |  |  | 215 - 2 nm | 0.85 | 0.20 | 1.58 | 99.48 |
|  | % RSD | | | 1.64 | 2.40 | 1.77 | 0.11 |
| MIC | Toluene (mL) | | 1 + 0.1 | 0.88 | 0.050 | ------ | 100.54 |
|  |  |  | 1 - 0.1 | 0.89 | 0.049 | ------ | 100.72 |
|  | % RSD | | | 0.80 | 1.43 | ------ | 0.13 |
|  | Triethylamine (mL) | | 0.3 + 0.05 | 0.85 | 0.040 | ------ | 100.87 |
|  |  |  | 0.3 - 0.05 | 0.87 | 0.042 | ------ | 100.44 |
|  | % RSD | | | 1.64 | 2.44 | ------ | 0.30 |
|  | Scanning wavelength (nm) | | 215 + 2 nm | 0.90 | 0.041 | ------ | 100.38 |
|  |  |  | 215 - 2 nm | 0.88 | 0.040 | ------ | 100.50 |
|  | % RSD | | | 1.59 | 1.75 | ------ | 0.09 |

^a^ Tailing factor and capacity factor determined for individual peak.

^b^ Resolution factor determined between each drug peak and the previous one.

^c^ % Assay was calculated from the regression equation.

**Figure S1: HPLC chromatograms for individual peaks of MET, NYS, and MIC.**


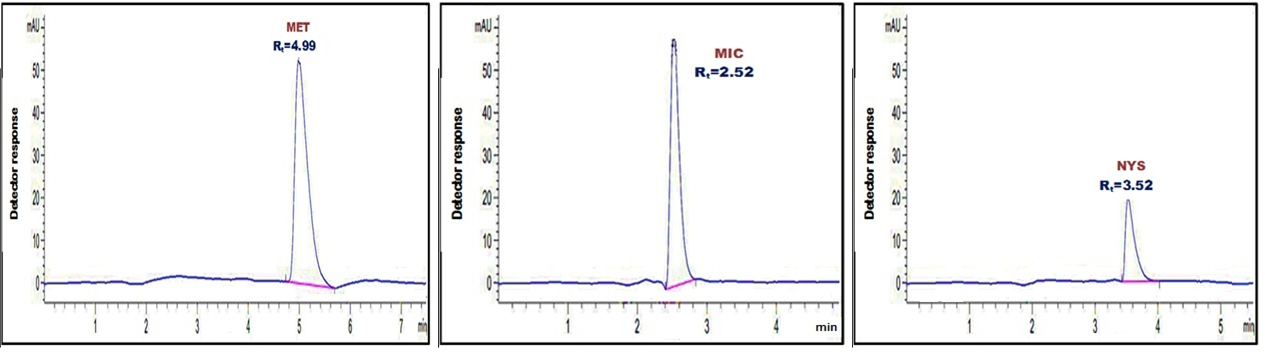


**Figure S2: TLC chromatograms for calibration curves for MET, NYS, and MIC.**


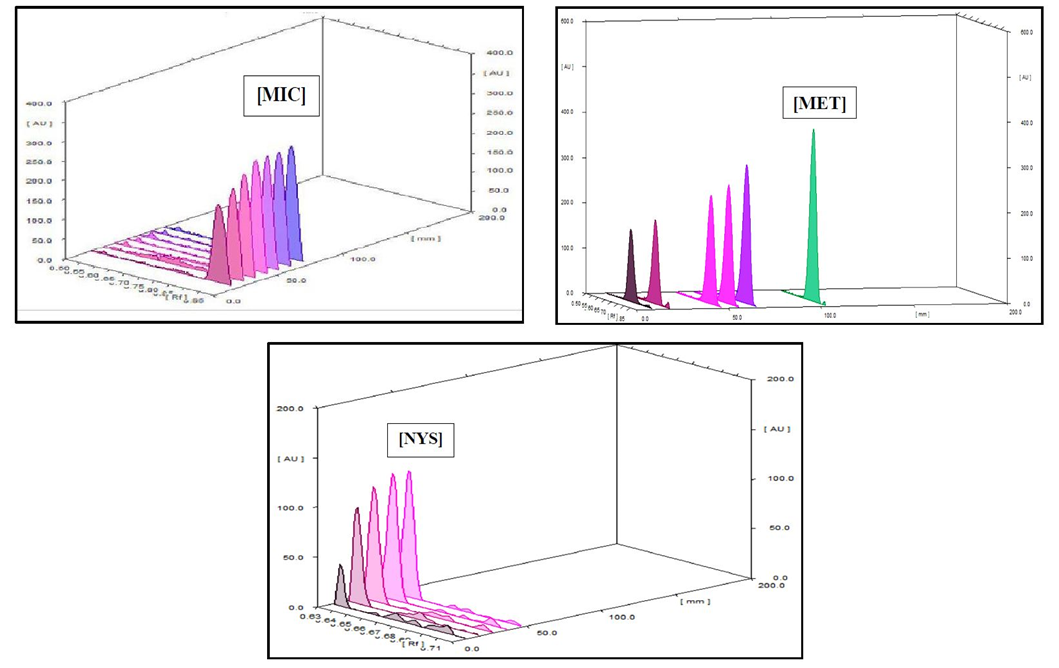

Supplement: Supplementary file 1 — Additional file 1: Table S1. Accuracy results as % recovery for determination of MIC, NYS and MET by the proposed HPLC and HPTLC methods. Table S2. Experimental results of robustness for determination of MIC, NYS and MET by the developed HPLC and HPTLC methods. Figure S1. HPLC chromatograms for individual peaks of MET, NYS, and MIC. Figure S2. TLC chromatograms for calibration curves for MET, NYS, and MIC. [file 13065_2023_1083_MOESM1_ESM.docx]
